# Correction: Regulatory rewiring drives intraspecies competition in *Bacillus subtilis*

**DOI:** 10.1371/journal.pgen.1012128

**Published:** 2026-04-21

**Authors:** Margarita Kalamara, Alistair Bonsall, Jonathan Griffin, Joana Carneiro, Marek Gierlinski, Lukas Eigentler, David Stevenson, Amy Wood, Michael Porter, Helge C. Dorfmueller, Cait E. MacPhee, James C. Abbott, Nicola R. Stanley-Wall

The following information is missing from the Data Availability statement: Raw sequencing reads and genome assemblies generated during this study have been deposited in the European Nucleotide Archive (ENA) under BioProject accession PRJEB98668.

## References

[1] Kalamara M, Bonsall A, Griffin J, Carneiro J, Gierlinski M, Eigentler L, et al. Regulatory rewiring drives intraspecies competition in Bacillus subtilis. PLoS Genet. 2026;22(2):e1012050. doi: 10.1371/journal.pgen.1012050 41701782 PMC12935306

