# Supplementary figures and images for: Regulatory rewiring drives intraspecies competition in Bacillus subtilis
Source: PLoS Genet. 2026 Feb 17;22(2):e1012050. doi: 10.1371/journal.pgen.1012050 (PMC12935306; doi:10.1371/journal.pgen.1012050)

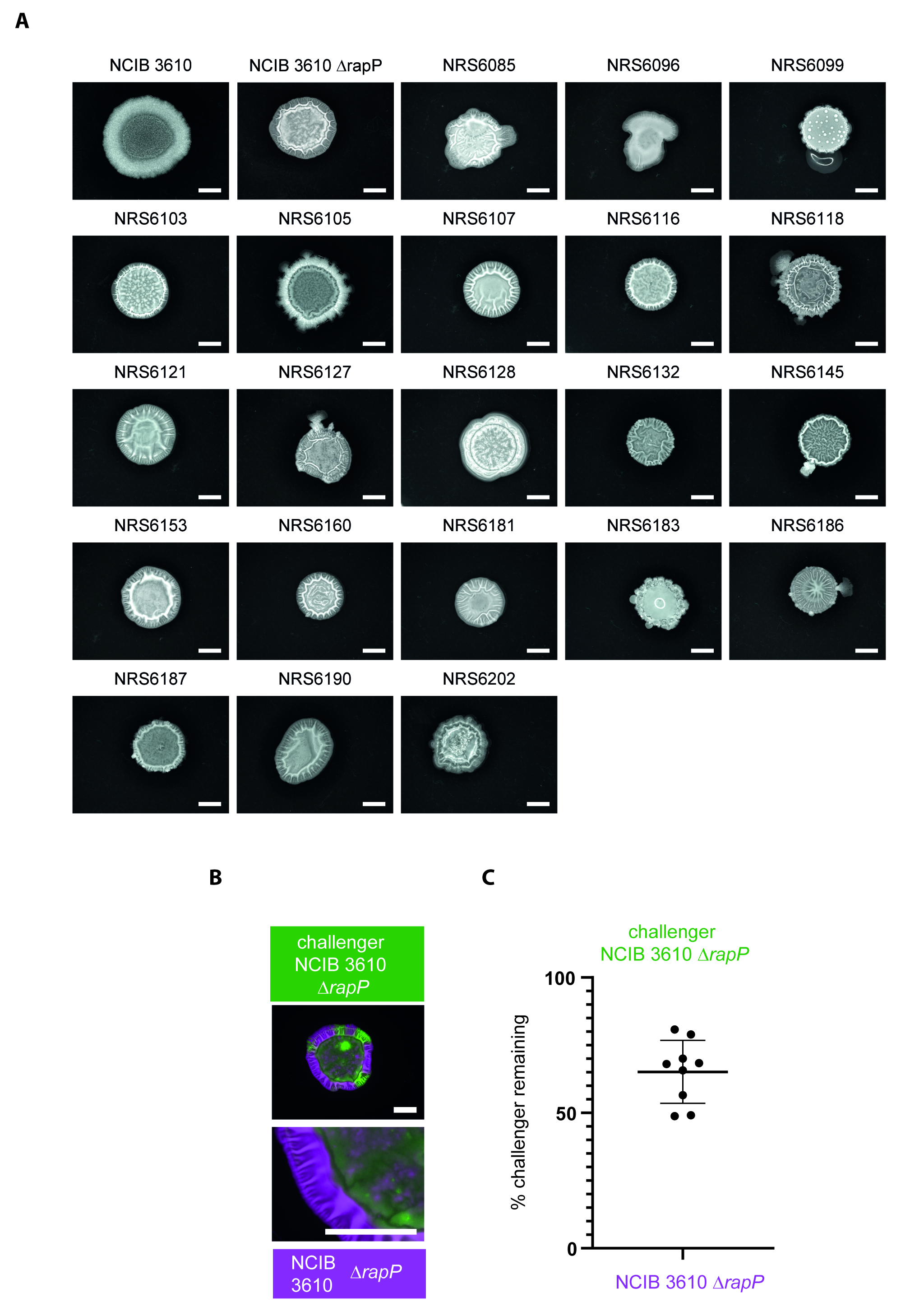

Supplement: S1 Fig — (A) Colony biofilm morphology of NCIB 3610 ΔrapP and other soil isolates of B. subtilis. Strains were grown on biofilm-inducing media for 48h at 30oC before imaging. The strain and genotype are indicated. The scale bars represent 0.5 cm. All soil isolates shown here are variants constitutively expressing GFP and the image of NCIB 3610 rapP is of an mTagBFP-expressing variant. (B) Representative outcome after 48 hours incubation in a dual isolate colony biofilm incubated at 30oC for 48 hours. (C) Quantification of the interaction outcome represented as %challenger remaining. The cocultured strain is indicated on the x-axis. Each data point represents a single colony biofilm and is derived from a combination of technical and biological repeats. The error bars represent the standard deviation of the mean. (TIF) [file pgen.1012050.s001.tif]

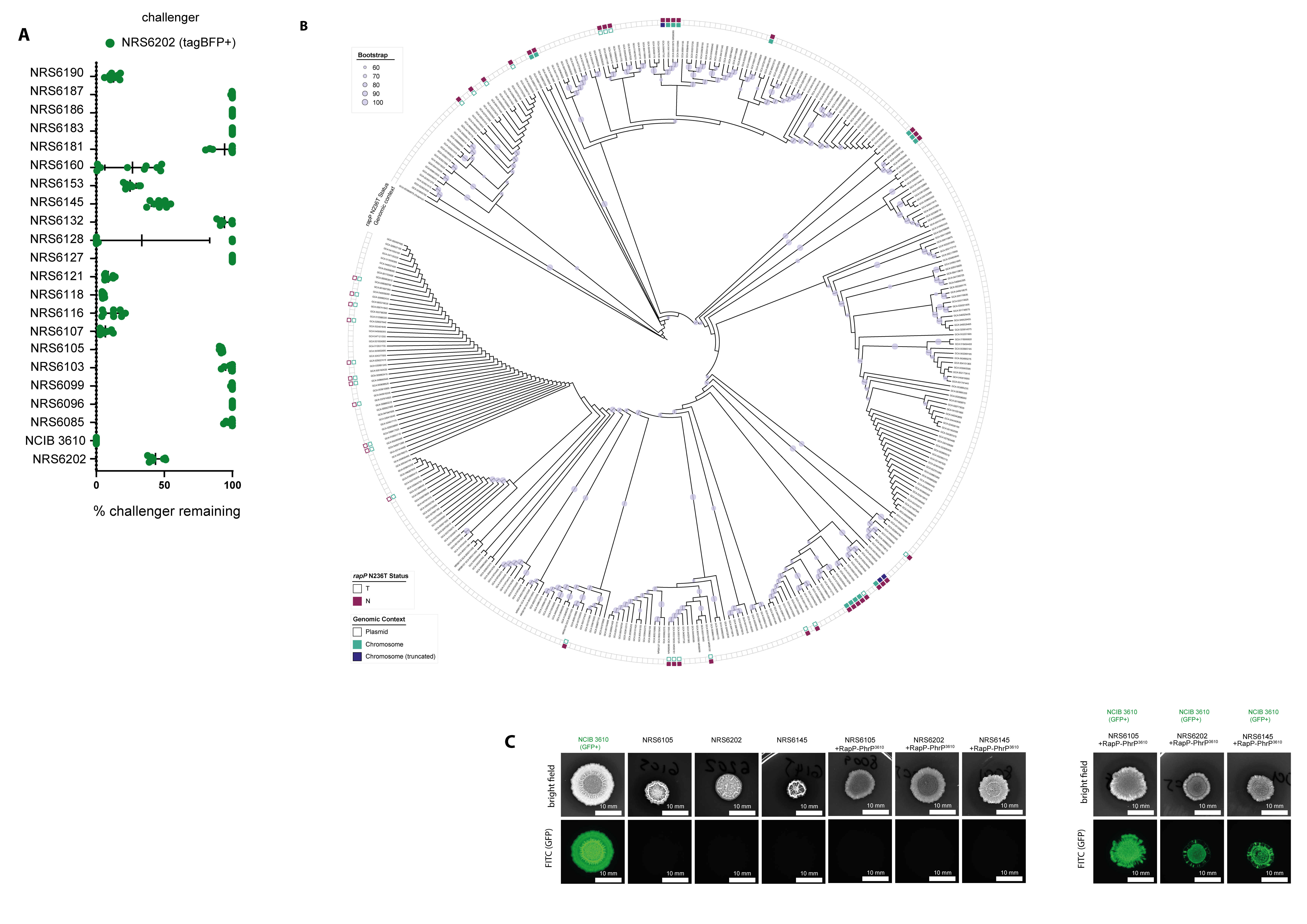

Supplement: S2 Fig — (A) Quantification of pairwise biofilm competitions shown as % challenger remaining. Challenger was NRS6202 (mTagBFP+). Partner strains are indicated on the y-axis. Each point = one biofilm; error bars = SD. (B) Prevalence of the rapP coding region in the genomes of 370 Bacillus subtilis isolates. B. subtilis isolates indicating isolates carrying rapP, and the state of the N236T residue associated with reduced signal responsiveness (C) Representative images of single isolate and dual isolate biofilms linked to Fig 2G. The scale bars represent 10 mm. The strains are as indicated. Images were taken after 48 hours incubation at 30oC. (TIF) [file pgen.1012050.s002.tif]

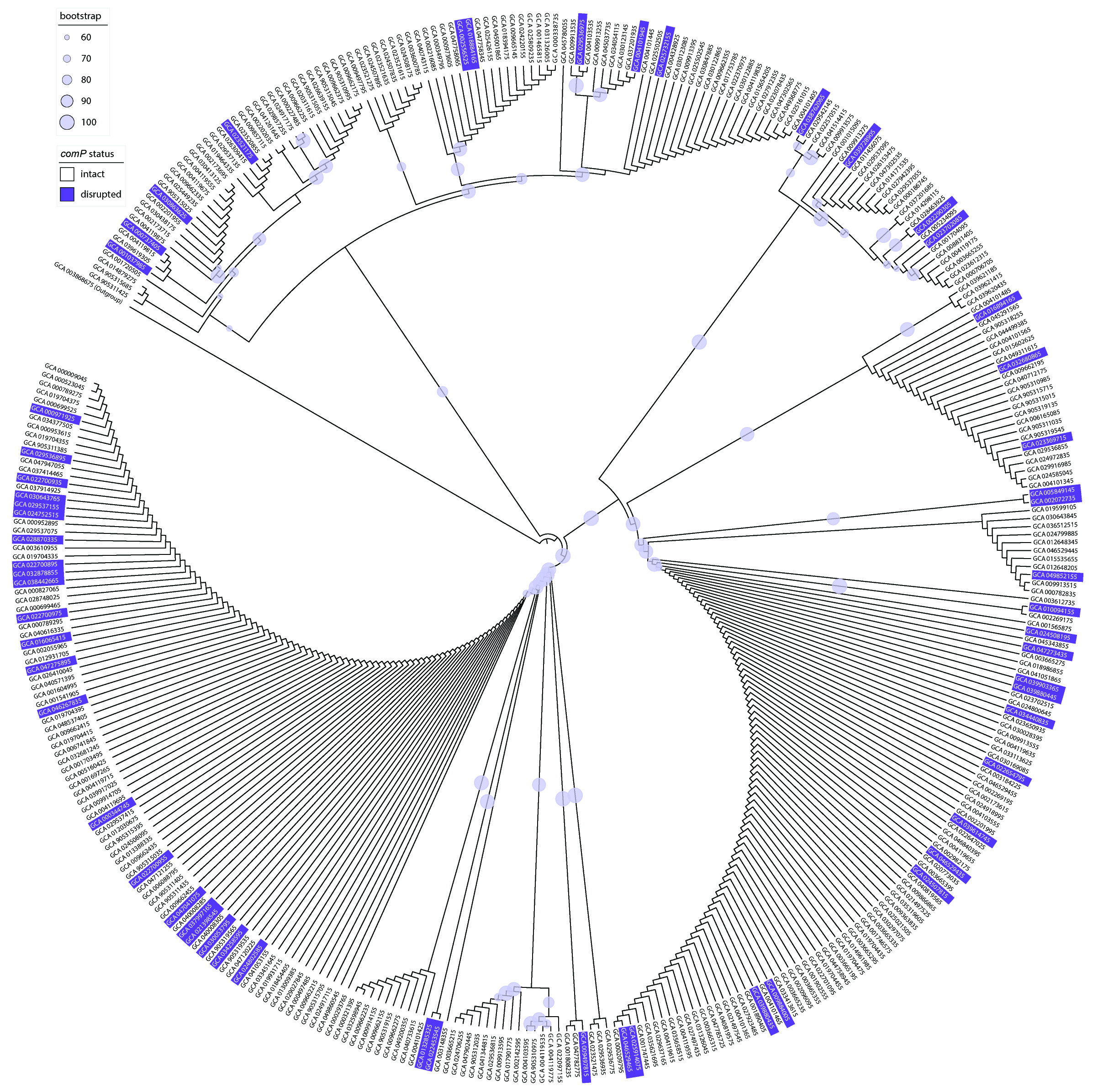

Supplement: S4 Fig — The comQ gene was selected as a conserved member of the comQXPA operon. The phylogeny is rooted on an outgroup consisting of a Bacillus amyloliquefaciens sequence. Bootstrap values are represented by differently sized circles on the tree branches. (TIF) [file pgen.1012050.s004.tif]

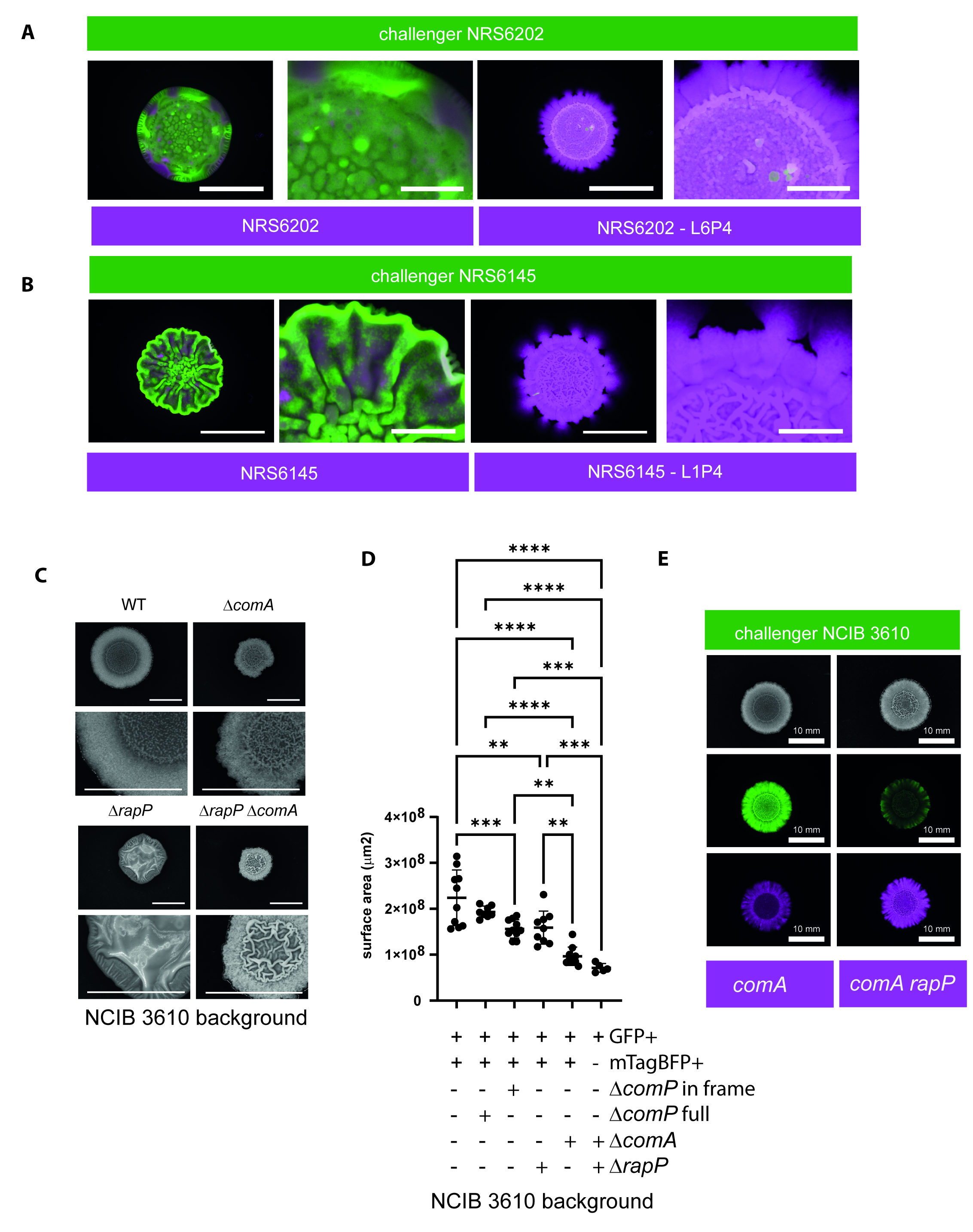

Supplement: S5 Fig — (A-B) Representative examples of colony biofilm interactions. The scale bars represent 10 mm or 3 mm. The strains in the coculture are indicated. Images were taken after 48 hours incubation at 30oC. (C) Colony biofilm morphology after growth on biofilm-inducing media for 48h at 30oC. The strain and genotype are indicated. All strains are GFP+ derivatives of NCIB 3610 (NRS6942, NRS7770, NRS7279 & NRS7771). The scale bars represent 1.0 cm. (D) Footprint measurements for colony biofilms of the genotypes detailed in the legend of the graph. Ordinary One-way ANOVA Tukey’s multiple comparisons test, with a single pooled variance, was used for analysis. The asterisks represent statistical significance with a p-value of ≤0.05 (*); ≤ 0.01 (**); ≤ 0.001 (***) or ≤0.0001 (****). (E) Colony biofilm morphology after growth on biofilm-inducing media for 48h at 30oC. The scale bars represent 10 mm. The strains in the coculture are indicated. (TIF) [file pgen.1012050.s005.tif]

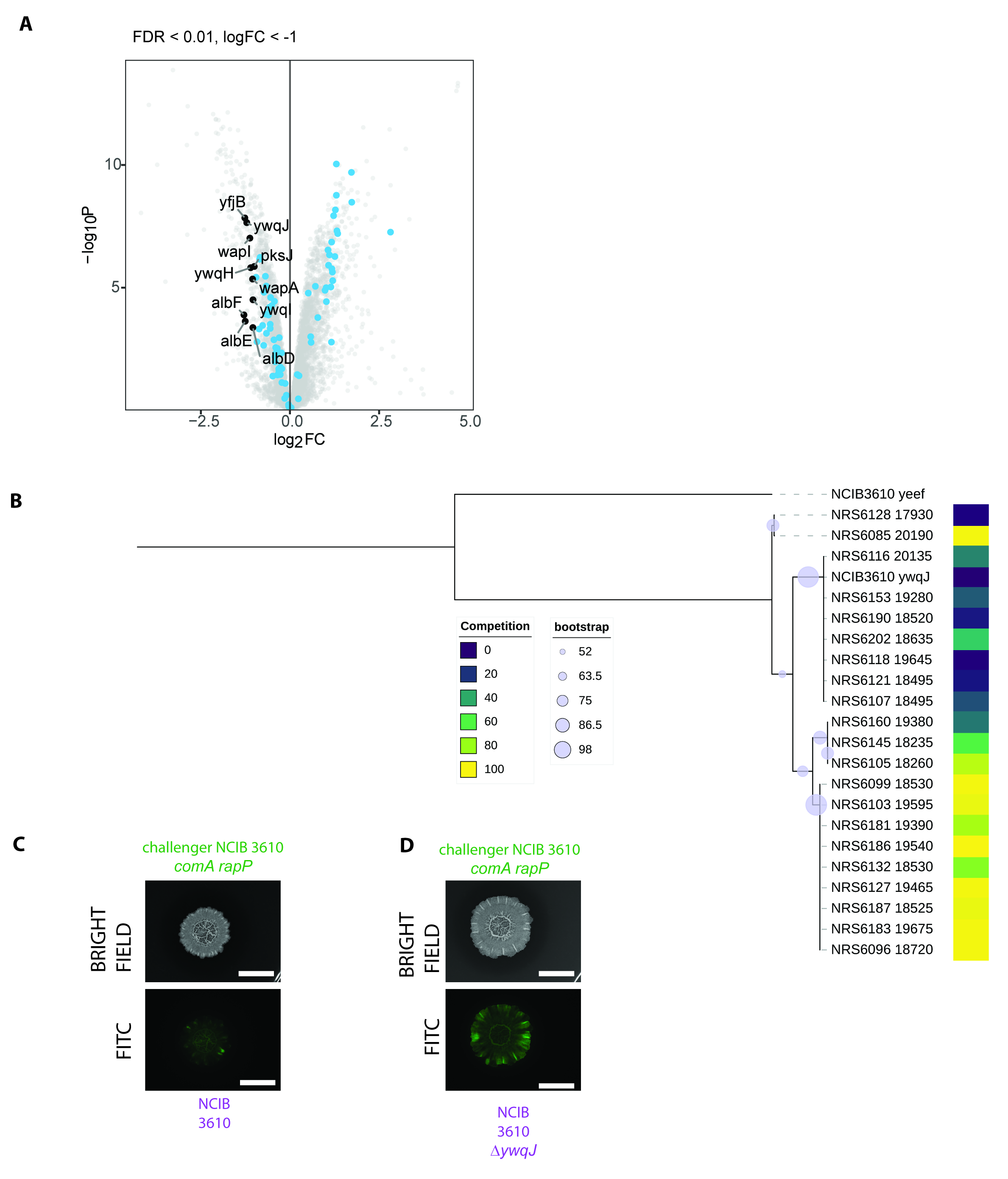

Supplement: S6 Fig — (A) Volcano plot of the 469 differentially expressed genes identified by RNA sequencing. The toxins and other antimicrobials that are expressed to a higher level in NCIB 3610 compared to the ΔrapP ΔcomA strain are indicated. (B) Phylogenetic tree based upon Muscle alignment of the top matched YwqJ LXG containing protein sequence from the suite of B. subtilis isolates relative YwqJ encoded by NCIB 3610. The LXG domain containing protein YeeF from NCIB 3610 was used as an outgroup. The heatmap represents the competition outcome of the different wild isolates when cocultured with NCIB 3610 ΔrapP which has an equivalent doubling time is shown (data from Fig 5K). (C) and (D) A representative outcome of a dual isolate colony biofilm morphology after growth on biofilm-inducing media for 48h at 30oC. The strains and genotypes are indicated. The upper panel is bright field, and the lower is the FITC signal false coloured green. The scale bars represent 1.0 cm. (TIF) [file pgen.1012050.s006.tif]
